# *Lactobacillus fermentum* Suo Attenuates HCl/Ethanol Induced Gastric Injury in Mice through Its Antioxidant Effects

**DOI:** 10.3390/nu8030155

**Published:** 2016-03-10

**Authors:** Huayi Suo, Xin Zhao, Yu Qian, Peng Sun, Kai Zhu, Jian Li, Baozhong Sun

**Affiliations:** 1 Institute of Animal Science, Chinese Academy of Agricultural Science, Beijing 100193, China; birget@swu.edu.cn; 2 College of Food Science, Southwest University, Chongqing 400715, China; 3 Department of Biological and Chemical Engineering, Chongqing University of Education, Chongqing 400067, China; zhaoxin@cque.edu.cn (X.Z.); qianyu@foods.ac.cn (Y.Q.); sunpeng@foods.ac.cn (P.S.); zhukai@foods.ac.cn (K.Z.); 4 Chongqing Collaborative Innovation Center of Functional Food, Chongqing University of Education, Chongqing 400067, China; 5 Chongqing Engineering Technology Research Center for Functional Food, Chongqing University of Education, Chongqing 400067, China; 6 Institute of Qinghai-Tibetan Plateau, Southwest University for Nationalities, Chengdu 610041, China

**Keywords:** *Lactobacillus fermentum* Suo, gastric injury, ethanol, antioxidant, mice

## Abstract

The purpose of the study was to determine the inhibitory effects of *Lactobacillus fermentum* Suo (LF-Suo) on HCl/ethanol induced gastric injury in ICR (Institute for Cancer Research) mice and explain the mechanism of these effects through the molecular biology activities of LF-Suo. The studied mice were divided into four groups: healthy, injured, LF-Suo-L and LF-Suo-H group. After the LF-Suo intragastric administration, the gastric injury area was reduced compared to the injured group. The serum MOT (motilin), SP (substance P), ET (endothelin) levels of LF-Suo treated mice were lower, and SS (somatostatin), VIP (vasoactive intestinal peptide) levels were higher than the injured group mice. The cytokine IL-6 (interleukin 6), IL-12 (interleukin 12), TNF-α (tumor necrosis factor-α) and IFN-γ (interferon-γ) serum levels were decreased after the LF-Suo treatment. The gastric tissues SOD (superoxide dismutase), GSH-Px (glutathione peroxidase), NO (nitric oxide) and activities of LF-Suo treated mice were increased and MDA (malondialdehyde) activity was decreased compared to the injured group mice. By the RT-PCR assay, LF-Suo raised the occludin, EGF (epidermal growth factor), EGFR (epidermal growth factor receptor), VEGF (vascular endothelial growth factor), Fit-1 (fms-like tyrosine kinase-1), IκB-α (inhibitor kappaB-α), nNOS (neuronal nitric oxide synthase), eNOS (endothelial nitric oxide synthase), Mn-SOD, Cu/Zn-SOD, CAT (catalase) mRNA or protein expressions and reduced the COX-2, NF-κB (nuclear factor kappaB), and iNOS (inducible nitric oxide synthase) expressions in gastric tissues compared to the gastric injured group mice. A high concentration (1.0 × 10^9^ CFU/kg b.w.) of LF-Suo treatment showed stronger anti-gastric injury effects compared to a low concentration of (0.5 × 10^9^ CFU/kg b.w.) of LF-Suo treatment. LF-Suo also showed strong survival in pH 3.0 man-made gastric juice and hydrophobic properties. These results indicate that LF-Suo has potential use as probiotics for its gastric injury treatment effects.

## 1. Introduction

Ethanol is the main ingredient used in Chinese spirits and alcoholic beverages and because of its fat-soluble nature, it easily causes stomach injury, so alcohol abuse can cause acute erosive hemorrhagic gastritis and long-term drinking could cause stomach disorders and chronic atrophic gastritis [1]. At high concentration of ethanol might cause stomach injury through direct erosion, and through metabolism ethanol converts to acetaldehyde, whose toxic effect may lead to stomach cancer [2]. Different studies explain alcoholic gastric damage mechanism; showing that oxygen free radicals and lipid peroxidation chain reaction is one of the important mechanisms in stomach injury. During ethanol metabolic processes in the body, neutrophils release oxygen free radicals, causing endothelial damage, microcirculation disturbance and inhibits gastric injury healing. Through respiratory burst, neutrophils continue to produce large amounts of oxygen free radicals, forming a vicious circle [3]. Hydrochloric acid and ethanol could cause imbalance between some endogenous aggressive factors and cytoprotective factors. HCl also could cause damage of gastric mucosa, and ethanol could reach the mucosa and induce cell rupture in the wall of blood vessels [4].

Malondialdehyde produced by oxygen free radical lipid peroxidation can react with DNA bases, inducing mutation damage [5]. Ethanol also leads to a decrease in antioxidant active constitutes, such as glutathione, in the body, raising ROS (reactive oxygen species) level and causing antioxidant enzyme inactivation and DNA chain rupture. Cell membrane lipid peroxidation damage causes release of lysosomal enzyme and aggravates gastric lesions. Control of ethanol oxidation and the level of oxygen free radicals in the body are important ways of reducing alcoholic stomach injury [6].

Yak, is a bovine animal, mainly seen in the Tibetan plateau of China, where yaks’ milk, meat and fur are all available. The amount of milk produced per day is lower with an average of 1.4–2.25 kg, but compared with common milk it contains higher nutrients such as protein, essential amino acids, minerals and so on [7]. Besides drinking, herdsmen on the Tibetan plateau use it to make yogurt. The difference between yak’s milk and ordinary milk, also due to its special geographic conditions, endows natural-fermented yak yogurt with special qualities. Its special quality was mainly due to the involvement of lactic acid bacteria during natural fermentation, and this team had isolated the lactic acid bacteria from yak yogurt. Early studies showed that lactic acid bacteria isolated from yak yogurt can better resist gastric acid and bile salt than *Lactobacillus bulgaricus*, which was commonly used in ordinary food industry [8]. Studies had shown that lactic acid bacteria isolated from yak yogurt had a stronger ability to survive in the stomach and intestines which could effectively play a vital role in human body. Meanwhile, the study on antioxidant effect of lactic acid bacteria isolated from yak yogurt showed that they all have antioxidant effects *in vitro*, and some high-quality lactic acid bacteria showed high antioxidant ability showing high potential for use [9]. Fujimura *et al.* [10] found that *lactobacillus* OLL2716 could stimulate *H. pylori*, the *lactobacillus* could make *H. pylori* retain dormancy, the growth of *H. pylori* was inhibited, and that this effect could protect the stomach. Another study also proved that *L. rhamnosus* GG could protect the stomach by the animal experiment, the lactobacillus could reduce the gastric mucosa cells apoptosis by increasing the PGE2 level, and this course inhibited the ethanol induced gastric mucosa damage [11]. *Lactobacillus* had many functional effects on peptic ulcer prevention and cure through gastric mucosal epithelial cell binding site competition, H. pylori colonization inhibiting, and inflammation related cytokine decreasing [12]. It has been shown that *Lactobacillus fermentum* Suo, a high-quality lactic acid bacteria strain used in this study, can inhibit gastric lesions through its antioxidant ability. The serum levels of MOL, SP, SS, VIP, ET, IL-6, IL-12, TNF-α, IFN-γ and tissue levels of SOD, GSH-Px, NO, MDA of mice were determined by molecular biology methods, such as serum kits and RT-PCR assay. The aim of the study was to determined the inhibitory effects of LF-Suo on gastric injury *in vivo* by observing mice stomach injury morphology, tissue damage and oxidation-related serum index, as well as changes of inflammatory cytokines in serum and the expression of oxidation and inflammation-related mRNA in stomach tissue. The further mechanism of anti-gastric injury effects checked through the anti-oxidation effects of LF-Suo were measured. These results and mechanism explanation will help to apply LF-Suo.

## 2. Materials and Methods

### 2.1. Microorganism Strains

*Lactobacillus fermentum* Suo was isolated and identified from yak yoghurt of Hongyuan grassland houseland (Hongyuan, Ngawa Prefecture of Sichuan Province, China) and deposited with the China Center for Type Culture Collection (CCTCC, Wuhan, China), bearing CCTCC Accession Number M2013511. *Lactobacillus bulgaricus* was purchased from Institute of Microbiology of the Chinese Academy of Sciences, Beijing, China.

### 2.2. Mice Experiment

The ICR mice (male, *n* = 40, 7 weeks old) were divided into four groups, these were healthy group, injured group, and low and high concentrations of *Lactobacillus fermentum* Suo group; there were 10 mice in each group. The healthy group and the injured group mice were only treated with 0.2 mL distilled water once a day by gavage for 14 days, the *Lactobacillus fermentum* Suo group were treated with 0.5 and 1.0 × 10^9^ CFU/kg b.w. concentrations of *Lactobacillus fermentum* Suo liquid by gavage for 14 days. Then all the mice were cut off from food for 24 h, but allowed to drink water freely. After 12 h the mice except for healthy mice were treated with the gastric injury inducing reagent (0.1 mL HCl/ethanol/10 g b.w., 60% in 150 mM HCl) by gavage [4]. All the mice were sacrificed by CO_2_ after induced gastric injury for 30 min, and the blood was collected and treated with centrifugal separation (4000 r/min, 10 min) for blood serum manufacture by high-speed temperature centrifuge (H2050R, Xiangyi centrifuge instrument Co., LTD, Changsha, Hunan, China). The experiments were performed following a protocol approved by the Animal Ethics Committee of Chongqing Medical University (Chongqing, China).

### 2.3. Mice Gastric Injury Evaluation

The gastric secretion volumes of mice were determined with a 10 mL measuring cylinder, and the pH levels of gastric juice of mice were determined using a Seven Easy pH meter (Mettler Toledo, Schwerzenbach, Switzerland). The isolated stomachs were inflated by injecting 1% formalin solution (10 mL) for 10 min to fix the tissues, and opened along the greater curvature. The area (mm^2^) of hemorrhagic lesions that had developed in the stomach was measured under a Leica MZ7.5 dissecting microscope (Leica, Bensheim, Germany) with a square grid. The gastric injury inhibitory rate (%) = (1 − gastric injury area of sample treated mice/gastric injury area of injured group mice) × 100 [13].

### 2.4. Mice Serum Levels Measurement

Serum MOT (motilin), SP (substance P), SS (somatostatin), VIP (vasoactive intestinal peptide) and ET (endothelin) levels were determined with radioimmunoassay kits (Beijing Puer Weiye Biotechnology Co., Ltd., Beijing, China) according to the manufacturer’s protocols.

### 2.5. Mice Cytokine IL-6, IL-12, TNF-*α* and IFN-γ Levels Measurement

Serum IL-6, IL-12, TNF-α and IFN-γ levels were measured with a commercial ELISA kit (ELISA MAX, Biolegend, San Diego, CA, USA) according to the manufacturer’s protocol.

### 2.6. Gastric Tissues SOD, GSH-Px, NO and MDA Activities Measurement

Gastric tissues SOD (superoxide dismutase), NO (nitric oxide) and MDA (malondialdehyde) activities were determined with the assay kits (Nanjing Jiancheng Bioengineering Institute, Nanjing, Jiangsu, China) according to the manufacturer’s protocols.

### 2.7. mRNA Expression Determination (RT-PCR Assay)

The mice gastric tissues were extracted from RNA by RNAzol reagent (GeneCopoeia Inc., Rockville, MD, USA). RNA extraction of gastric tissues is diluted to 1 μg/μL. Then the 1 μL of oligod T18, RNase, dNTP, MLV (murine leukemia virus) enzymes and 10 μL of 5 × buffer were added into the 2 μL RNA extraction of gastric tissues to synthesize cDNA under the conditions of 37 °C for 120 min, 99 °C for 4 min, 4 °C for 3 min. Then the occludin, EGF, EGFR, COX-2, VEGF, NF-κB, IκB-α, Mn-SOD, Cu/Zn-SOD, CAT and GAPDH expressions were amplified by the method of reverse transcription-polymerase chain reaction. Sequences of primers used to specifically amplify the genes of interest were as in Table 1. PCR was then carried out in an automatic thermocycler (Bioneer, Daej-eon, South Korea) for 30 cycles (94 °C for 30 s, 55 °C for 30 s, and 72 °C for 40 s) followed by an 8-min extension at 75 °C. Agarose gel (1%) with ethidium bromide was used for electrophoresis to check the PCR amplification products [14].

### 2.8. Protein Expression Determination (Western Blot Assay)

Protein lysates were added into the gastric tissues after rinsing with pre-cooled PBS 3 times, lysed at 4 °C and centrifuged (10,000 r/min) for 15 min. Supernatant proteins were then extracted and mixed with SDS-PAGE (sodium dodecyl-sulfate-polyacrylamide gelelectrophoresis) loading buffer. Primary antibodies were put into them after SDS-PAGE gel electrophoresis and transfer to a membrane, and the proteins were placed overnight at 4 °C. Then horseradish peroxidase-conjugated secondary antibodies were incubated with the proteins at room temperature. Finally, immunoreactive proteins were tested with a chemiluminescent enhanced chemiluminescence assay kit (GE Healthcare, Uppsala, Sweden) and observed with a LAS3000 luminescent image analyzer (Fujifilm, Tokyo, Japan) with β-actin as internal reference [8].

### 2.9. Endurance Capacity of LF-Suo to pH3.0 Manmade Gastric Juice

The artificial gastric juice was made by 0.2% of NaCl and 0.35% of pepsin, adjusted to pH 3.0 and then vacuum-filtered to remove bacteria in the clean bench. 5 mL of reactivated bacteria culture was centrifuged at 3000 rpm for 10 min, and the bacteria pellet was collected and re-suspended in 5 mL of sterile saline. The suspension (1 mL) was mixed with 9 mL of the artificial gastric juice and incubated in thermostatic oscillator at 37 °C and 300 rpm. A sample volumes of 200 μL was pipetted at 0 and 3 h and plates were poured with MRS (de Man, Rogosa and Sharpe) agar and incubated at 37 °C for 48 h. CFU (Colony Forming Units) was counted and the survival rate was determined [15].

### 2.10. Determination the Hydrophobic Property of LF-Suo

A 5 mL sample of reactivated bacteria culture was centrifuged at 3000 rpm for 10 min. The bacterial pellet was collected and re-suspended in 5 mL of PBS (phosphate buffer saline) buffer (50 mM, pH 6.5), and the suspension was centrifuged at 3000 rpm for 10 min; the process was repeated once again. Using PBS buffer as the blank of absorption, the final bacteria suspension was adjusted by PBS buffer to make a 1.00 absorbance (denoted as A_0_) at 560 nm. 4 mL of the adjusted bacteria suspension was added with 0.8 mL of dimethylbenzene, vibrated for 30 s and then placed for stratification. The aqueous layer was measured for the absorbance (denoted as A) at 560 nm (blank: PBS buffer) and the results were recorded [15].

### 2.11. Statistical Analysis

Differences between the mean values for individual groups were assessed by one-way analysis of variance (ANOVA) with Duncan’s multiple range test *p* < 0.05 was considered to indicate a statistically significant difference. SAS version 9.2 (SAS Institute Inc., Cary, NC, USA) was used to conduct the statistical analyses.

## 3. Results

### 3.1. Stomach Appearances of Mice

**Figure 1:**
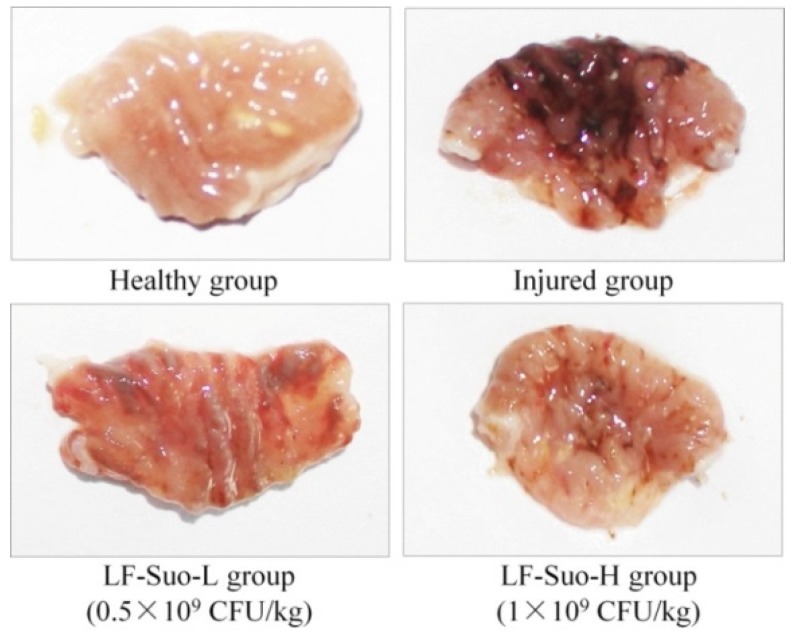
Stomachs of studied mice.

After HCl/ethanol treatment, the injuries in mouse stomachs were observed by stomach photo assay (Figure 1). The stomachs of healthy group mice showed no injury and the surface of gastric mucosa was clean. The gastric injury area of stomach in the injured group mice was the highest LF-Suo reduced the gastric injury; the high concentration of LF-Suo treated mice showed smaller injury area than lower concentration of LF-Suo (Table 2).

LF-Suo-L, *Lactobacillus fermentum* Suo (0.5 × 10^9^ CFU/kg b.w.); LF-Suo-H, *Lactobacillus fermentum* Suo (1.0 × 10^9^ CFU/kg b.w.).

### 3.2. Gastric Secretion Volume and pH of Gastric Juice of Mice

As shown in Table 3, gastric secretion volume of injured group mice was highest, but the pH of gastric juice was lowest. The healthy group mice showed reversed trends. LF-Suo decreased the gastric secretion volume and increased the pH of the gastric juice compared to the injured group.

### 3.3. Serum MOT (Motilin), SP (Substance P), SS (Somatostatin), VIP (Vasoactive Intestinal Peptide) and ET (Endothelin) Levels in Mice

LF-Suo-H increased SS, VIP serum levels and decreased MOT, SP, ET levels compared to the injured group mice, and the SS, VIP levels of LF-Suo-H treated mice were only higher than healthy group, the MOT, SP, ET levels also lower than healthy group (Table 4).

### 3.4. Cytokine IL-6, IL-12, TNF-*α* and IFN-*γ* Levels in Mice

The healthy mice had the lowest cytokine IL-6, IL-12, TNF-α and IFN-γ levels, and HCl/ethanol treatment (injured group) raised these levels (Table 5). LF-Suo inhibited these increasing levels, the increased effects of LF-Suo-H were stronger than LF-Suo-L.

### 3.5. Gastric Tissues SOD, GSH-Px, NO and MDA Activities of Mice

The gastric tissue levels of SOD, GSH-Px, NO and MDA were determined by kits (Table 6), the SOD, GSH-Px, NO activities were highest in healthy group mice and the MDA activity was lowest. The SOD, GSH-Px, NO activities of LF-Suo-H treated mice were higher than LF-Suo-L group and injured group mice, the MDA activity showed the opposite trends.

### 3.6. mRNA and Protein Expression of Occludin and COX-2 in Gastric Tissues of Mice

**Figure 2:**
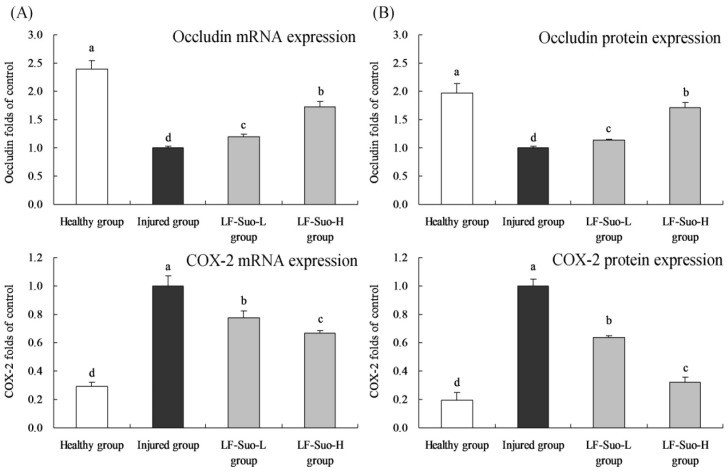
Occludin, COX-2 mRNA (**A**) and protein (**B**) expression of studied mice. Fold-ratio: gene expression/GAPDH (β-actin) × injured group numerical value (injured group fold ratio: 1). ^a–d^ Mean values with different letters in the same column are significantly different (*p* < 0.05) according to Duncan’s multiple-range test.

The RT-PCR and western blot assay results showed that LF-Suo increased the occludin mRNA and protein expressions in gastric tissues of mice compared to the injured group mice (Figure 2), but LF-Suo decreased the COX-2 expressions. LF-Suo-H treatment showed the higher mRNA and protein occludin (1.73 and 1.72 folds of injured group) expressions than LF-Suo-L (1.19 and 1.13 folds of injured group) treatment, and LF-Suo-H showed lower mRNA and protein COX-2 (0.32 and 0.67 folds of injured group) expressions than LF-Suo-L (0.64 and 0.78 folds of injured group).

LF-Suo-L, *Lactobacillus fermentum* Suo (0.5 × 10^9^ CFU/kg b.w.); LF-Suo-H, *Lactobacillus fermentum* Suo (1.0 × 10^9^ CFU/kg b.w.).

### 3.7. mRNA Expression of EGF and EGFR in Gastric Tissues of Mice

**Figure 3:**
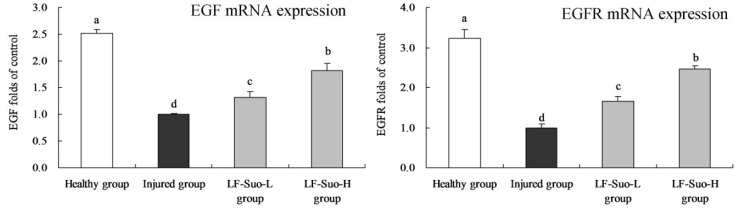
EGF and EGFR mRNA expression of studied mice. Fold-ratio: gene expression/GAPDH × injured group numerical value (injured group fold ratio: 1). ^a–d^ Mean values with different letters in the same column are significantly different (*p* < 0.05) according to Duncan’s multiple-range test. LF-Suo-L, *Lactobacillus fermentum* Suo (0.5 × 10^9^ CFU/kg b.w.); LF-Suo-H, *Lactobacillus fermentum* Suo (1.0 × 10^9^ CFU/kg b.w.).

After using different concentration of LF-Suo treatment, the EGF and EGFR mRNA expressions in gastric tissues of gastric injury mice were significantly increased (*p* < 0.05, Figure 3). The highest concentrations of LF-Suo (LF-Suo-H) were associated with EGF (1.81 folds of injured group) and EGFR (2.47 folds of injured group) expressions at high levels.

### 3.8. mRNA Expression of VEGF and Fit-1 in Gastric Tissues of Mice

**Figure 4:**
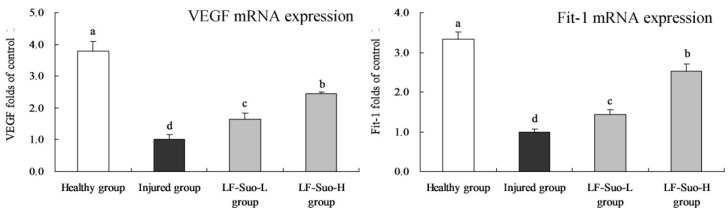
VEGF, Fit-1 mRNA expression of studied mice. Fold-ratio: gene expression/GAPDH × injured group numerical value (injured group fold ratio: 1). ^a–d^ Mean values with different letters in the same column are significantly different (*p* < 0.05) according to Duncan’s multiple-range test. LF-Suo-L, *Lactobacillus fermentum* Suo (0.5 × 10^9^ CFU/kg b.w.); LF-Suo-H, *Lactobacillus fermentum* Suo (1.0 × 10^9^ CFU/kg b.w.).

LF-Suo-H raised the VEGF (2.45 folds of injured group) and Fit-1 (2.53 folds of injured group) mRNA expressions compared to the injured group group (Figure 4), and these expressions were higher than LF-Suo-L group, but lower than the healthy group.

### 3.9. mRNA Expression of NF-*κ*B and I*κ*B-*α* in Gastric Tissues of Mice

**Figure 5:**
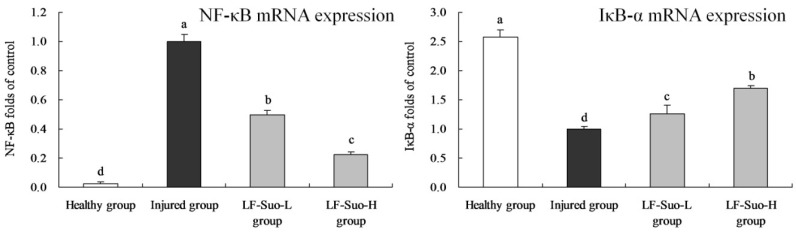
NF-κB, IκB-α mRNA expression of studied mice. Fold-ratio: gene expression/GAPDH × injured group numerical value (injured group fold ratio: 1). ^a–d^ Mean values with different letters in the same column are significantly different (*p* < 0.05) according to Duncan’s multiple-range test. LF-Suo-L, *Lactobacillus fermentum* Suo (0.5 × 10^9^ CFU/kg b.w.); LF-Suo-H, *Lactobacillus fermentum* Suo (1.0 × 10^9^ CFU/kg b.w.).

The NF-κB mRNA expression of gastric injured group mice was strongest and IκB-α expression was weakest (Figure 5). These expressions of healthy mice showed the opposite trends compared to the injured group. LF-Suo-H treated mice had higher IκB-α (1.69 folds of injured group) expression than LF-Suo-L (1.25 folds of injured group) and injured group mice, and had lower NF-κB (0.22 folds of injured group) expressions.

### 3.10. mRNA and Protein Expression of nNOS, eNOS and iNOS in Gastric Tissues of Mice

**Figure 6:**
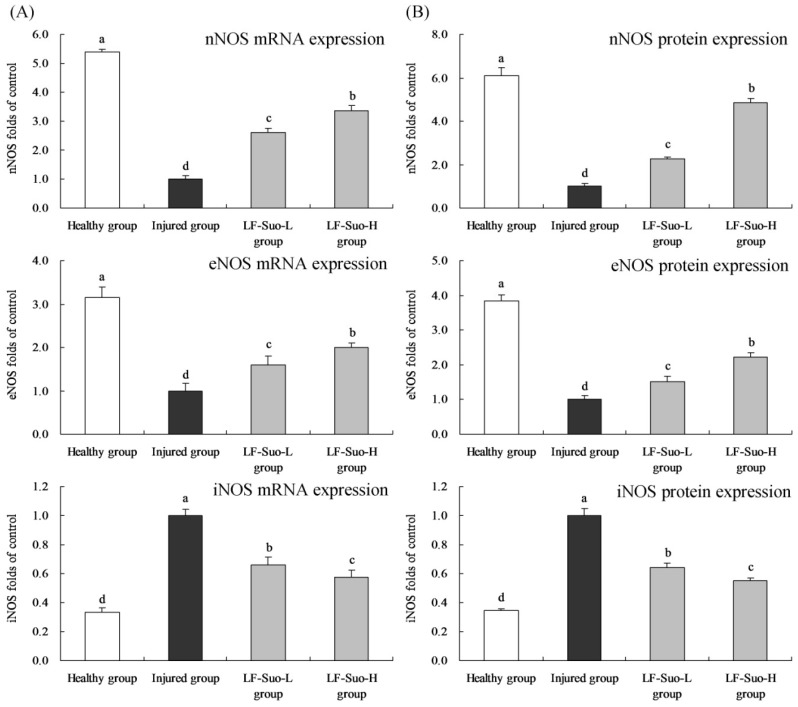
nNOS, eNOS, iNOS mRNA (**A**) and protein (**B**) expression of studied mice. Fold-ratio: gene expression/GAPDH (β-actin) × injured group numerical value (injured group fold ratio: 1). ^a–d^ Mean values with different letters in the same column are significantly different (*p* < 0.05) according to Duncan’s multiple-range test. LF-Suo-L, *Lactobacillus fermentum* Suo (0.5 × 10^9^ CFU/kg b.w.); LF-Suo-H, *Lactobacillus fermentum* Suo (1.0 × 10^9^ CFU/kg b.w.).

The LF-Suo-L, LF-Suo-H treated mice and health showed the higher nNOS, eNOS and lower iNOS mRNA and protein expressions than injured group mice (Figure 6). LF-Suo-H mice had 3.36 and 4.84 folds nNOS, 2.00 and 2.22 folds eNOS, 0.58 and 0.55 folds iNOS mRNA and protein expressions of injured group.

### 3.11. mRNA and Protein Expression of Mn-SOD, Cu/Zn-SOD and CAT in Gastric Tissues of Mice

**Figure 7:**
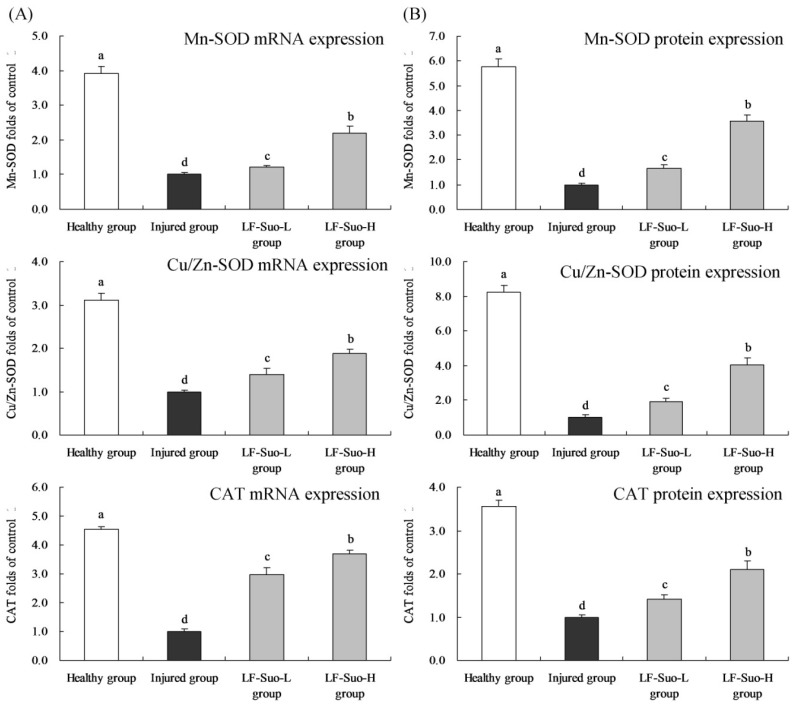
Mn-SOD, Cu/Zn-SOD, CAT mRNA (**A**) and protein (**B**) expression of studied mice. Fold-ratio: gene expression/GAPDH (β-actin) × injured group numerical value (injured group fold ratio: 1). ^a–d^ Mean values with different letters in the same column are significantly different (*p* < 0.05) according to Duncan’s multiple-range test. LF-Suo-L, *Lactobacillus fermentum* Suo (0.5 × 10^9^ CFU/kg b.w.); LF-Suo-H, *Lactobacillus fermentum* Suo (1.0 × 10^9^ CFU/kg b.w.).

As shown in Figure 7, the Mn-SOD, Cu/Zn-SOD and CAT mRNA and protein expressions of gastric tissues in healthy mice were highest, the LF-Suo-H treated mice also had high levels of Mn-SOD (2.21 and 3.58 folds of injured group), Cu/Zn-SOD (1.87 and 4.05 folds of injured group) and CAT (3.70 and 2.10 folds of injured group) mRNA and protein expressions.

### 3.12. In Vitro Properties of Lactobacillus Fermentum Suo

By the *in vitro* experiments, the survival in pH 3.0 manmade gastric juice and hydrophobic property of LF-Suo were 90.87% ± 5.28% and 71.65% ± 4.33%. LF-Suo showed a strong survival in pH 3.0 manmade gastric juice and hydrophobic property *in vitro*, in the past date of our publication, these *in vitro* activities of LF-Suo were stronger than common lactic acid bacteria of *Lactobacillus bulgaricus* [8].

## 4. Discussion

Animal experiments show that through direct observation after stomach injury, gastric mucosa tissues showed festering, and detecting a festered area could determine the degree of the stomach damage [13]. Ethanol promotes the expression of H^+^-K^+^-ATP in gastric mucosa and increases the secretion of gastric acid and pepsin, which is one of the important factors of gastric mucosa injury. When ethanol causes stomach hemorrhagic lesions, mucosal blood flow decreases, Na^+^ pumps out and K^+^ pumps in, causing gastric acid rebound. So present treatment of ethanol gastric mucosa damage is to reduce gastric acid secretion [16]. The international standard requires that bacteria concentration of lactic acid bacteria drink must be higher than 10^7^ CFU/mL, the lactic acid bacteria concentration of 10^9^ CFU/mL was also used for experiment in rats [11]. In our past research, the 10^9^ CFU/kg dose LF-Suo had a good preventive effect on constipation [8], this concentration amount to about 10^7^ CFU/mL for human. Based on these studies, we also chose 0.5 × 10^9^ CFU/kg and 1.0 × 10^9^ CFU/kg for this study. The healthy group mice without induced tissue injury were used as the healthy condition comparison in experiments. The healthy group mice had low MOT, SP, ET, IL-6, IL-12, TNF-α, IFN-γ serum levels and high SS, VIP levels, these trends were also similar to the healthy population [3,13].

MOT and SP are excitatory gastrointestinal hormones. Stimulated by stomach injury, the levels of MOT and SP became higher and secretion of gastric acid increases greatly, which makes the internal stomach acidic and aggravates gastric lesions [14]. SS and VIP are inhibitory gastrointestinal hormones, which could inhibit the secretion of stomach acid [17]. Animal experiments showed that lowering the levels of MOT and SP and raising the level of SS and VIP could significantly inhibit gastric lesions [18]. ET could reduce gastric mucosal blood flow, weakening the protection of gastric mucosa, while ET could also affect contraction of gastric smooth muscle and increase stomach movement, which had side effects in the process of gastric lesions; gastric injury could raise the ET level [19].

Controlling the content of pro-inflammatory factors in the body was also an effective way of inhibiting gastric lesions. Animal experiments showed that ethanol causes the increase of cytokines such as TNF-α, IFN-γ, IL-6, IL-12 in gastric mucosa [14]. A study found that probiotics could limit the production of TNF-α and IFN-γ and increase the concentration of sIgA [20]. By increasing sIgA, gastric mucosa epithelium reduces apoptosis, maintains epithelial barrier function, and reduces inflammatory reaction caused by ethanol. Ethanol causes the release of oxygen free radicals, promoting inflammation [21]. And COX-2 could also play a role in defense by inhibiting pro-inflammatory function [22].

After gastric damage, some tissues showed oxidation phenomenon. As important antioxidant enzymes, SOD and GSH-Px could convert peroxide caused by stomach tissue oxidation to low-poisonous or harmless substance, which was beneficial to the recovery of gastric damage [22]. The imbalance between stomach tissue damage and protection factors relatively lowered its defense capability, causing stomach injury. Gastric mucus could prevent gastric mucosa from digestive enzymes and external stimuli, which was a crucial defense factor of gastric mucosa. Excessive drinking (drinking equal to 60 mL absolute alcohol once) could significantly reduce mucus thickness and content of amino hexose in gel layer and reduce mucosal defense capability [23]. NO could protect gastric mucosa and increase gastric mucosal blood flow. The content of NO declined significantly in patients with gastric ulcer, so NO was proven to be an active component of gastric ulcer prevention [24]. MDA was the marker of oxidative stress and generated in great quantities after gastric tissue injury, which could be used as an index of gastric ulcer [25].

The GAPDH (glyceraldehyde-3-phosphate dehydrogenase) gene shows high expression in almost all body tissues; GAPDH gene expression is generally invariable in homologous cells or tissue when it is used as housekeeping gene, therefore GAPDH gene is used as an internal control for PCR assay [26]. β-actin protein is also generally invariable in homologous cells or tissues of mammals; β-actin antibody is used for western blot assay by its internal control effect [27]. Occludin protein was one of the most important parts in close connection and played a key role to maintain the structure of close connection and membrane barrier. In mucosa epithelium, when occludin expression changed, reduced and missed, mucosal barrier showed dysfunction and mucous membrane permeability increased, causing gastric mucosa injury [28]. Microcirculation disturbance was one of the reasons for the damaged gastric mucosa barrier. As vasodilation factors, NO and PGE2 could inhibit platelet aggregation and thrombosis and promote gastric mucosa microcirculation, synthesis of protein and cell renewal to regulate immune function and improve the ability to repair mucous membrane, COX-2 was a important factor to synthesize PGE2 [29]. ET was the strongest vasoconstrictor in the body. Ethanol could inhibit the synthesis and secretion of NO and PGE2 to reduce mucosal blood flow. And through the release of histamine, small arteries expanded and capillary permeability increased, causing mucosal microcirculation. Then lipid peroxide in tissue and oxygen free radicals increased, which promoted the release of ET and causes serious damage of gastric mucosa [30]. A study has shown that PGE2 could also inhibit the level of TNF-α and IFN-γ *in vivo* [31], these effects might be increased by an increase in COX-2 levels.

Mast cells infiltration plays an important role in ethanol-induced ulcer. Mast cells infiltration causes release of histamine in large quantities, and histamine is closely related to lipid peroxidation. Antioxidants could inhibit lipid peroxidation and improve the activity of antioxidant enzymes, which had been regarded as an adjuvant treatment for alcoholic gastric mucosa damage [32]. EGF as an antioxidant could stabilize mast cells, while probiotics could inhibit ethanol-induced lipid peroxidation, reduced gastric damage and promoted healing of gastric injury [33]. EGF is the ligand of EGFR, which could combine and activate EGFR as autocrine or paracrine growth factors to induce EGFR phosphorylation, providing continuous division signals to cells and causing cell proliferation and differentiation [34]. Studies have shown that EGF and EGFR both are expressed in normal gastric mucosa parietal cells, and that the EGFR autocrine system plays an advantageous role in gastric mucosa epithelium metabolism and maintaining structural integrity [35]. EGF and EGFR could inhibit the secretion of gastric acid, and their autocrine system plays an important role in cell proliferation and differentiation during ulcer healing [36].

VEGF acted on vascular endothelial cells and blood vessels and participates in inflammation and damage repair, which was also an important indicator to inspect tissue growth and wound healing. Stomach damage and its healing mechanism were closely related to VEGF [37]. Experiments demonstrated that single dose VEGF lavage could prevent ethanol-caused acute gastric mucosa damage, which could stimulate new blood vessel formation three weeks later and promote the healing of peptic ulcers [38]. The VEGF family has three receptors, including VEGFR-l, VEGFR-2 and VEGFR-3, respectively encoded by the Fit-1, Flk-1-KDR and Fit-4 genes [39]. By increasing the expression of VEGF and Fit-1, tissues regenerated and diluted harmful substances to protect gastric mucosa [40].

COX-2 is an important inflammatory factor, which could inhibit inflammation in gastric damage and significantly alleviate stomach injury. Controlling the expression of COX-2 in gastric tissue could inhibit stomach inflammation [41]. Meanwhile, COX-2 and its product prostaglandin could adjust factors such as VEGF and EGFR. As a source, COX-2 could activate VEGF and EGFR in downstream and promote gastric lesions [42]. Under normal conditions, NF-κB existed in compound proteins in cell plasma, and after activation could move into the nucleus and combine with double-stranded DNA to activate transcription and expression of many downstream genes [43]. IKK was activated by external stimuli and made IκB phosphorylation degrade and dissociate with NF-κB. Then NF-κB was activated and transferred into the nucleus to regulate gene transcription [44]. NF-κB is very important in the generation of gastritis, and its expression was higher in gastric damage than that in normal state, while the expression of IκB-α was lower. Mucosal inflammation stimulated by NF-κB might be the key to influence stomach injury, showing that degradation of IκB-α could inhibit the activation of NF-κB [45].

Many forms of enzymes are related to prostaglandins and NO metabolism [46]. NOS exists both in basic and induced forms, and basic form NOS includes eNOS in endothelial cells and nNOS in intestinal neurons [47]. After proper stimulation such as endotoxin, NOS could convert into induced iNOS [48]. Under physiological condition, eNOS and nNOS continuously synthesized NO to regulate stomach tissues. iNOS was not expressed in the normal state, but was activated after stomach tissue injury, intensifying stomach injury. In the process of alleviating stomach injury, increasing the content of eNOS and nNOS and lowering the content of iNOS could effectively play a positive role [49].

SOD can convert superoxide free radicals into hydrogen peroxide, and in mammalian tissues, there are three kinds of SOD isozymes, including Cu/Zn-SOD (SOD1), Mn-SOD (SOD2) and EC-SOD (SOD3) [50]. Large amounts of Cu/Zn-SOD exist in the cytoplasm, nucleus, and peroxidase and mitochondrial membranes, whose basic function is to reduce the concentration of O_2_^−^ in cells and protect epithelial cells. Mn-SOD is a mitochondrial enzyme and exists in the mitochondrial matrix, which could clear O_2_^−^ produced by respiratory chain reactions to prevent peroxidation damage [51]. Through antioxidant effects, Cu/Zn-SOD and Mn-SOD could inhibit alcoholic gastric lesions. SOD plays a vital role in balancing oxidation and antioxidants in the body, because SOD can remove O_2_^−^ by disproportionation reaction to protect cells from damage [52]. CAT removes oxide free radicals by deoxygenizing H_2_O_2_ to H_2_O and prevented the generation of OH to terminate the radical chain reaction. As antioxidant enzymes, SOD and CAT had been proven to be able to protect gastric mucosa [53]. In previous research, LF-Suo had strong anti-gastric juice effects and hydrophobic properties, LF-Suo had stronger viability in stomach than *Lactobacillus bulgaricus* [8]. The high survival rate of lactic acid bacteria in gastric acid means the lactic acid bacteria might be used as probiotics [54], and the mucosa adsorptivity of lactic acid bacteria was in relationship with its hydrophobic property; the high hydrophobic property might help lactic acid bacteria to adsorb in gastric mucosa. These characteristics might make LF-Suo colonize in stomach and show anti-gastric injury effect.

LF-Suo is a new lactic acid bacteria which was found in yak yoghourt; its *in vitro* effects have been investigated, but there are not many studies about its *in vivo* functional effects; there is one report on its anti-constipation determination [8]. In this study, the gastric injury treatment effects of LF-Suo were measured and its possible mechanism discussed. These data indicate that this lactic acid bacterial species has gastric injury inhibitory effects similar to those found in others [10,11,12].

## 5. Conclusions

After intra-gastric administration of LF-Suo, the gastric injury area was reduced, and the inhibitory rate was raised. The serum MOT, SP, ET levels of LF-Suo treated mice were reduced, and SS, VIP levels were raised compared to that of the injured group mice. The serum cytokine IL-6, IL-12, TNF-α and IFN-γ levels in LF-Suo treated mice were lower than in the injured group mice. The SOD, GSH-Px, NO activities in gastric tissues of LF-Suo treated mice were increased and MDA activity was decreased compared to the injured group mice. LF-Suo increased the occludin, nNOS, eNOS, Mn-SOD, Cu/Zn-SOD, CAT mRNA and protein expressions and decreased the COX-2, iNOS expressions in gastric tissues compared to the injured group in mice. And LF-Suo also raised the EGF, EGFR, VEGF, Fit-1, IκB-α mRNA expressions and reduced the NF-κB expression compared to the injured group in mice. The higher concentration of LF-Suo (LF-Suo-H) treatment showed the better anti-gastric injury effects. These results indicate that LF-Suo could be used for probiotics or functional food manufacture for gastric injury preventation through its antioxidant effects.

## Figures and Tables

**Table 1:** Sequences of reverse transcription-polymerase chain reaction primers used in this study.

Gene Name	Sequence
Occludin	Forward: 5′-CTGTCTATGCTCGTCATCG-3′
	Reverse: 5′-CATTCCCGATCTAATGACGC-3′
COX-2	Forward: 5′-TTA AAA TGA GAT TGT CCG AA-3′
	Reverse: 5′-AGA TCA CCT CTG CCT GAG TA-3′
EGF	Forward: 5′-GCC AAG CTC AGA AGG CTA C-3′
	Reverse: 5′-CAG GCC AGC CTC GTC TCA T-3′
EGFR	Forward: 5′-TCG GTG CTG TGC GAT TTA-3′
	Reverse: 5′-TTT CTG GCA GTT GCT CCT C-3′
VEGF	Forward: 5′-GCA CCC ATG GCA GAA GGA GGA G-3′
	Reverse: 5′-GTG CTG ACG CTA ACT GAC C-3′
Fit-1	Forward: 5′-CAA GTG GCC AGA GGC ATG GAG TT-3′
	Reverse: 5′-GAT GTA GTC TTT ACC ATC CTG TTG-3′
NF-κB	Forward: 5′-CAC TTA TGG ACA ACT ATG AGG TCT CTG G-3′
	Reverse: 5′-CTG TCT TGT GGA CAA CGC AGT GGA ATT TTA GG-3′
IκB-α	Forward: 5′-GCT GAA GAA GGA GCG GCT ACT-3′
	Reverse: 5′-TCG TAC TCC TCG TCT TTC ATG GA-3′
nNOS	Forward: 5′-GAA TAC CAG CCT GAT CCA TGG AA-3′
	Reverse: 5′-TCC TCC AGG AGG GTG TCC ACC GCA TG-3′
eNOS	Forward: 5′-GGA GAG GCT GCA TGA CAT TG-3′
	Reverse: 5′-GGT AGA GCC ATA GTG GAA TGA C-3′
iNOS	Forward: 5′-AGA GAG ATC GGG TTC ACA-3′
	Reverse: 5′-CAC AGA ACT GAG GGT ACA-3′
Cu/Zn-SOD	Forward: 5′-GAAGAGAGGCATGTTGGAGA-3′
	Reverse: 5′-CCAATTACACCACGAGCCAA-3′
Mn-SOD	Forward: 5′-TTCAATAAGGAGCAGGGAC-3′
	Reverse: 5′-CAGTGTAAGGCTGACGGTTT-3′
CAT	Forward: 5′-AGATACTCCAAGGCGAAGGTG-3′
	Reverse: 5′-AAAGCCACGAGGGTCACGAAC-3′
GAPDH	Forward: 5′-CGG AGT CAA CGG ATT TGG TC-3′
	Reverse: 5′-AGC CTT CTC CAT GGT CGT GA-3′

**Table 2:** Stomach appearance of studied mice.

Group	Gastric Injury	
	Gastric Injury Area (mm^2^) ^1^	Inhibitory Rate (%) ^1^
Healthy group	0.0 ± 0.0 ^d^	100 ± 0.0 ^a^
Injured group	7.63 ± 0.41 ^a^	0.0 ± 0.0 ^d^
LF-Suo-L group	4.37 ± 0.24 ^b^	42.7 ± 2.8 ^c^
LF-Suo-H group	2.18 ± 0.26 ^c^	71.4 ± 3.1 ^b^

^1^ Values are mean ± SD. ^a–d^ Mean values with different letters in the same column are significantly different (*p* < 0.05) according to Duncan’s multiple-range test. LF-Suo-L, *Lactobacillus fermentum* Suo (0.5 × 10^9^ CFU/kg b.w.); LF-Suo-H, *Lactobacillus fermentum* Suo (1.0 × 10^9^ CFU/kg b.w.).

**Table 3:** Gastric secretion volume and pH of studied mice.

Group	Gastric Secretion Volume (mL) ^1^	pH of the Gastric Juice ^1^
Healthy group	0.30 ± 0.02 ^d^	3.5 ± 0.1 ^a^
Injured group	1.39 ± 0.28 ^a^	1.1 ± 0.2 ^d^
LF-Suo-L group	0.88 ± 0.09 ^b^	2.3 ± 0.2 ^c^
LF-Suo-H group	0.51 ± 0.05 ^c^	2.9 ± 0.2 ^b^

^1^ Values are mean ± SD. ^a–d^ Mean values with different letters in the same column are significantly different (*p* < 0.05) according to Duncan’s multiple-range test. LF-Suo-L, *Lactobacillus fermentum* Suo (0.5 × 10^9^ CFU/kg b.w.); LF-Suo-H, *Lactobacillus fermentum* Suo (1.0 × 10^9^ CFU/kg b.w.).

**Table 4:** Serum MOT, SP, SS, VIP and ET levels in studied mice.

Group	MOT (μg/L) ^1^	SP (μg/L) ^1^	SS (μg/L) ^1^	VIP (μg/L) ^1^	ET (μg/L) ^1^
Healthy group	61.6 ± 4.7 ^d^	52.6 ± 3.8 ^d^	109.3 ± 7.3 ^a^	93.2 ± 6.2 ^a^	11.2 ± 0.5 ^d^
Injured group	141.7 ± 11.6 ^a^	116.2 ± 8.1 ^a^	60.7 ± 4.2 ^d^	54.1 ± 3.4 ^d^	20.3 ± 1.2 ^a^
LF-Suo-L group	106.3 ± 5.9 ^b^	86.8 ± 4.3 ^b^	77.6 ± 2.8 ^c^	67.0 ± 2.7 ^c^	17.5 ± 0.4 ^b^
LF-Suo-H group	84.5 ± 3.0 ^c^	72.6 ± 2.2 ^c^	89.3 ± 3.1 ^b^	74.3 ± 2.1 ^b^	15.2 ± 0.6 ^c^

^1^ Values are mean ± SD. ^a–e^ Mean values with different letters in the same column are significantly different (*p* < 0.05) according to Duncan’s multiple-range test. LF-Suo-L, *Lactobacillus fermentum* Suo (0.5 × 10^9^ CFU/kg b.w.); LF-Suo-H, *Lactobacillus fermentum* Suo (1.0 × 10^9^ CFU/kg b.w.).

**Table 5:** Cytokine IL-6, IL-12, TNF-α and IFN-γ levels of studied mice.

Group	IL-6 (pg/mL) ^1^	IL-12 (pg/mL) ^1^	TNF-α (pg/mL) ^1^	IFN-γ (pg/mL) ^1^
Healthy group	43.6 ± 2.4 ^d^	246.3 ± 31.9 ^d^	45.6 ± 2.6 ^d^	42.0 ± 2.2 ^d^
Injured group	128.7 ± 8.1 ^a^	877.9 ± 48.3 ^a^	141.2 ± 7.8 ^a^	93.6 ± 6.8 ^a^
LF-Suo-L group	88.6 ± 3.9 ^b^	522.8 ± 34.6 ^b^	87.3 ± 5.5 ^b^	79.4 ± 2.4 ^b^
LF-Suo-H group	65.3 ± 2.7 ^c^	461.7 ± 36.0 ^c^	70.1 ± 3.3 ^c^	63.9 ± 3.1 ^c^

^1^ Values are mean ± SD. ^a–d^ Mean values with different letters in the same column are significantly different (*p* < 0.05) according to Duncan’s multiple-range test. LF-Suo-L, *Lactobacillus fermentum* Suo (0.5 × 10^9^ CFU/kg b.w.); LF-Suo-H, *Lactobacillus fermentum* Suo (1.0 × 10^9^ CFU/kg b.w.).

**Table 6:** Gastric tissues SOD, GSH-Px, NO and MDA activities of studied mice.

Group	SOD (U/mgprot) ^1^	GSH-Px (U/mgprot) ^1^	NO (μmol/gprot) ^1^	MDA (nmol/mgprot) ^1^
Healthy group	86.2 ± 5.2 ^a^	142.8 ± 3.7 ^a^	6.1 ± 0.4 ^a^	2.9 ± 0.2 ^e^
Injured group	47.6 ± 2.5 ^d^	108.3 ± 5.0 ^d^	1.7 ± 0.2 ^d^	6.7 ± 0.3 ^d^
LF-Suo-L group	55.8 ± 2.6 ^c^	120.8 ± 4.5 ^c^	3.2 ± 0.3 ^c^	4.5 ± 0.3 ^c^
LF-Suo-H group	68.1 ± 2.2 ^b^	129.7 ± 5.2 ^b^	4.8 ± 0.2 ^b^	3.9 ± 0.1 ^b^

^1^ Values are mean ± SD. ^a–e^ Mean values with different letters in the same column are significantly different (*p* < 0.05) according to Duncan’s multiple-range test. LF-Suo-L, *Lactobacillus fermentum* Suo (0.5 × 10^9^ CFU/kg b.w.); LF-Suo-H, *Lactobacillus fermentum* Suo (1.0 × 10^9^ CFU/kg b.w.).
